# Role of beta-2-microglobulin as a biomarker in very preterm and extremely preterm infants with CNS inflammation

**DOI:** 10.1371/journal.pone.0216498

**Published:** 2019-05-07

**Authors:** Albert Batista Muñoz, Stephanie Hadley, Marti Iriondo Sanz, Thais Agut Quijano, Marta Camprubí Camprubí

**Affiliations:** 1 BCNatal | Barcelona Center for Maternal Fetal and Neonatal Medicine, Hospital Sant Joan de Déu and Hospital Clínic, University of Barcelona, Esplugues de Llobregat, Spain; 2 Vanderbilt University School of Medicine, Nashville, Tennessee, United States of America; Universidad Miguel Hernandez de Elche, SPAIN

## Abstract

**Background:**

Premature infants are at risk for severe sepsis and meningitis, both infections associated with high mortality and morbidity. Cerebro-spinal fluid (CSF) culture is the gold standard method for meningitis diagnosis, but interpretation of biochemical parameters of CSF is essential at the moment of the analysis in order to start the appropriate treatment. The main objective of this study was to determine whether levels of CSF beta-2-microglobulin (B2M) were elevated in preterm infants with CNS infections or other inflammatory processes, and to establish if there were differences in B2M concentrations amongst various inflammatory settings (sepsis, meningitis, and progressive post-hemorrhagic ventricular dilatation (PHVD)).

**Methods:**

This is a retrospective study of all very preterm and extremely preterm infants (< 32 weeks of gestation) admitted to our NICU between 2012 and 2017. All those who underwent a lumbar puncture during their stay as part of a sepsis work-up or PHVD were considered for inclusion. CSF biochemical parameters and B2M were tested in all of the patients.

**Results:**

Fifty-nine patients were included in the study. In patients with CNS infections, the median value of B2M was 8.69 mg/L (3.92–18.5). B2M levels above 3.92 mg/L showed greater sensitivity and specificity than leukocyte levels in discriminating between patients with CNS infections or other inflammatory processes and those without CNS inflammation.

**Conclusions:**

In this population, CSF B2M proved to be an effective biomarker to discriminate between patients with CNS infections and other inflammatory processes and those without CNS inflammation.

## Introduction

Premature infants are at increased risk of sepsis and meningitis [1]. Five percent of very preterm and extremely preterm infants evaluated for sepsis are subsequently diagnosed with meningitis, so a lumbar puncture (LP) is mandatory when sepsis is suspected [2]. Sepsis and meningitis are serious infections associated with high mortality and morbidity [3]. Severe inflammation in an immature brain, such as in very preterm infants, can have harmful effects on long-term development. As a result, early recognition and treatment are essential to improve survival and decrease the risk of long-term sequelae [4]. In our institution, when sepsis is suspected, blood, cerebrospinal fluid (CSF) and urine cultures are performed, and then antibiotics are started. While CSF culture is the gold standard method for diagnosing meningitis, it often takes several days to produce a final result. Therefore, the correct interpretation of biochemical CSF parameters upon initial analysis is essential in order to start appropriate treatment in a timely manner.

Reference values of white blood cells (WBC), CSF protein and glucose in the CSF of healthy term newborns are widely described in the literature [5,6,7]. However, interpreting biochemical CSF values in very preterm and extremely preterm infants is difficult because, in many cases, there are confounding factors such as traumatic LPs, previous antibiotic use and/or the presence of intraventricular hemorrhage (IVH) [5].

To improve the utility of LPs, several cytokines have been used to discriminate between bacterial and viral infections [8, 9]. However, routine measurements of these CSF biomarkers are still not universally available.

Beta-2-microglobulin (B2M) is a low molecular weight protein (11800D) that constitutes the light chain of the class I major histocompatibility complex [10]. Its presence in biological fluids reflects a rate of cell membrane renovation and cellular turnover, and there is increasing evidence that it is a useful inflammatory biomarker in many infectious, autoimmune and neoplastic central nervous system (CNS) disorders [11,12].

High CSF B2M levels have been reported in neonates with CNS infections [13,14]. However, like CSF WBC reference values, there is limited literature reporting normal values of B2M in preterm babies compared to term babies.

With this study, we sought to assess CSF B2M concentrations in very preterm and extremely preterm infants in the setting of different clinical conditions.

The objectives of this study were: (1) to determine whether levels of CSF B2M were elevated in very preterm and extremely preterm infants with CNS infections or other inflammatory processes, and (2) to determine if there were differences in CSF B2M concentrations between various inflammatory settings (sepsis, meningitis, and progressive post-hemorrhagic ventricular dilatation (PHVD)) in very preterm and extremely preterm infants.

## Material and methods

### Study population

Medical records of all very preterm (28–32 weeks of gestation) and extremely preterm infants (<28 weeks of gestation) who were admitted to the NICU at Hospital Sant Joan de Déu, Barcelona between 2012 and 2017 were reviewed. All preterm infants who underwent an LP during their stay as part of a septic work-up or PHVD evaluation were considered for inclusion. Patients with traumatic lumbar punctures, defined as the presence of >500 red blood cells (RBC)/mm^3^ in the CSF in patients without intracerebral hemorrhage, were excluded.

Data were obtained from the medical records of enrolled subjects and stored in an electronic database. Information collected included demographics, vital signs, birth histories, and other clinical variables related to the NICU clinical course.

### Patient classification

Included subjects were classified into three groups based on the final diagnosis and the reason for the LP:

#### Group 1 (control group)

Patients with normal CSF and no CNS infection. In these patients, an LP was performed to rule out infection because of clinical or analytical suspicion or positive blood culture. All of these patients had negative CSF cultures and polymerase chain reactions (PCRs), WBC < 20mm3, and normal glucose and protein levels.

#### Group 2 (meningitis group)

Patients with pleocytosis (>20 WBC/mm^3^) and/or microbiologic confirmation of a CSF infection based on a positive CSF culture or PCR.

#### Group 3 (post-hemorrhagic ventricular dilatation (PHVD) group)

Patients with PHVD who underwent CSF sampling via LPs or from ventricular reservoirs. None of the patients in this group had CNS infections.

### Definitions of variables

**Traumatic lumbar puncture** was defined as a CSF RBC count >500 /mm^3^ in patients without an intracerebral hemorrhage.

**Bacterial meningitis** was defined as the isolation of a bacterial pathogen in the CSF. Because CSF sterilization may have occurred if antibiotics were administered before the lumbar puncture, bacterial meningitis was defined as the presence of bacteria on CSF gram-stain with low CSF glucose or elevated CSF protein values.

**Bacteremia** was defined as the isolation of a bacterial pathogen in the blood; isolates that reflected commensal skin flora, such as coagulase-negative staphylococci, were considered contaminants [5].

**CSF pleocytosis** was defined as a CSF WBC count >20/mm3 (for infants aged ≤28 days) or >15/mm3 (for infants aged 29–56 days).

**Post-hemorrhagic ventricular dilatation** was defined as either a rapid severe increase in lateral ventricle size on cranial ultrasound within seven days of IVH or the continued progression of ventricular dilatation identified on cranial ultrasound for more than 14 days after IVH, characterized by a change in qualitative classification. PHVD was monitored using ventricular index and anterior horn width [15].

**CNS inflammation** was defined as increased WBCs in the CNS due to an infection or an inflammatory process after an IVH.

### CSF analysis

CSF evaluations included a standard cytochemical analysis with quantification of WBC, RBC, protein and glucose levels, and quantification of B2M concentration. In our center, B2M concentration is routinely performed in newborn CSF evaluations. All biochemical CSF parameters, including B2M levels, are reported within two hours.

CSF gram-stains and cultures were performed. If a viral infection was suspected, viral PCR was also performed.

### Statistical analysis

Continuous variables were described using mean, median, SD and range. The comparison between numerical variables was performed by an analysis of variance test (one-way ANOVA) and Kruskal-Wallis test. The Spearman correlation method was used to calculate the correlation between continuous variables. Multivariable analysis was performed and logistic regressions were used. ROC curves were obtained to evaluate the efficacy of the tests. Statistical significance was met when the P value was less than 0.05. Statistical analyses were processed with statistical software STATA13.

### Ethical aspects

All data used in this study were collected from patient medical records and de-identified. Informed consent was not obtained because the study was retrospective. Declaration of Helsinki (Fortaleza version) was considered for the research. The project was approved by the local Ethics Committee (Hospital Sant Joan de Déu, Barcelona, Spain)

## Results

### Patient characteristics

Of 73 very preterm and extremely preterm infants with at least one CSF B2M measurement, 6 were excluded due to an unclear diagnosis of meningitis and 8 were excluded due to traumatic lumbar puncture. Mean gestational age at birth was 27.25 weeks with a range between 23–32 with a mean birth weight of 1026.37g with a range of 430g-1780g. The main clinical characteristics of our patients are included in *Table 1*.

**Table 1:** Patients characteristics.

	Control group (n = 36)	CNS infection (n = 9)	PHVD (n = 14)	P-value
**Gender (Male)**	**61.1% (n = 22)**	**33.3% (n = 3)**	**28.7% (n = 4)**	**0.074**
**Gestational age**	**27.6 (2.49)** **28 [26.58–29]**	**27.3 (1.65)** **27 [26.02–28]**	**26.1 (2.31)** **26 [24–27.37]**	**0.1293**
**Birthweight**	**1084 [584–1780]**	**934.7 [430–1500]**	**947 [440–1580]**	**0.3185**
**Surfactant administration**	**45.71% (n = 16)**	**66.66% (n = 6)**	**85.71% (n = 12)**	**0.022**
**PDA**	**62.1% (n = 23)**	**55.55% (n = 5)**	**85.71% (n = 12**	**0.1515**
**ROP**	**37.14% (n = 13)**	**42.85% (n = 4)**	**83.83% (n = 11)**	**0.0195**
**NEC**	**13.8% (n = 5)**	**0% (n = 0)**	**15.38% (n = 3)**	**0.4907**
**Intestinal Perforation**	**5% (n = 2)**	**0% (n = 0)**	**14.2% (n = 2)**	**0.3937**
**IVH**	**25% (n = 9)**	**66.6% (n = 6)**	**100% (n = 14)**	**0.0001**
**Mortality**	**5% (n = 2)**	**22.2% (n = 2)**	**21.4% (n = 3)**	**0.1788**

CNS: Central nervous System; PHVD: progressive post-hemorrhagic ventricular dilatation PDA: Persistent ductus arteriosus; ROP: retinopathy of prematurity; NEC: necrotizing enterocolitis; IVH: intraventricular hemorrhage

Results are expressed in Mean (Standard deviation), Median [95% confidence interval], or percentage.

There were 59 CSF samples. In 45 patients, an LP was performed to rule out sepsis. The remaining 14 patients suffered from PHVD, and therefore an LP was performed to reduce CSF pressure. Six of these patients with PHVD had ventricular reservoir tapping following an LP.

### General laboratory CSF characteristics

WBC, glucose, protein and B2M in CSF were significantly different between the 3 groups (p<0.05) (*Table 2*).

**Table 2:** CSF characteristics.

	Control group (n = 36)	CNS infection (n = 9)	PHVD (n = 14)	P-value
**WBC** **(n/mm3)**	8.77 (6.93) 10 [5–10]	2263.3 (2500.2) 800 [181.16–4849]	98.60 (137) 60 [11.18–159.36]	0.0001
**Proteins** **(mg/dL)**	130 (51.8) 128 [100.9–144]	403.44 (359.78) 316 [161.17–720]	226.9 (111.3) 217 [146.09–286.87]	0.0001
**Glucose** **(mg/dL)**	65.5(25.10) 59 [53.16–67]	34.2(18.4) 32 [16.31–56.22]	32.21(28.2) 27 [13.83–50.65]	0.002
**B2M** **(mg/L)**	2.81 (0.78) 2.62 [2.36–3.15]	9.03 (5) 6.97 [5.34–15.46]	5.53(2.05) 5.46 [4.47–6.98]	0.001

CNS: Central nervous System; PHVD: progressive post-hemorrhagic ventricular dilatation; WBC: white blood cells; RBC: red blood cells

Results are expressed in Mean (Standard deviation) and Median [95% confidence interval].

There was an increase in WBC and protein levels in the CNS infection group when compared to the others (p = 0.001). PHVD and CNS infection groups exhibited significantly higher CSF B2M levels when compared to the control group (p = 0.0001). In the post-hoc analysis, CSF B2M levels between PHVD and CNS infection groups were also different (p = 0.02), with the highest levels in infected patients.

**Fig 1:**
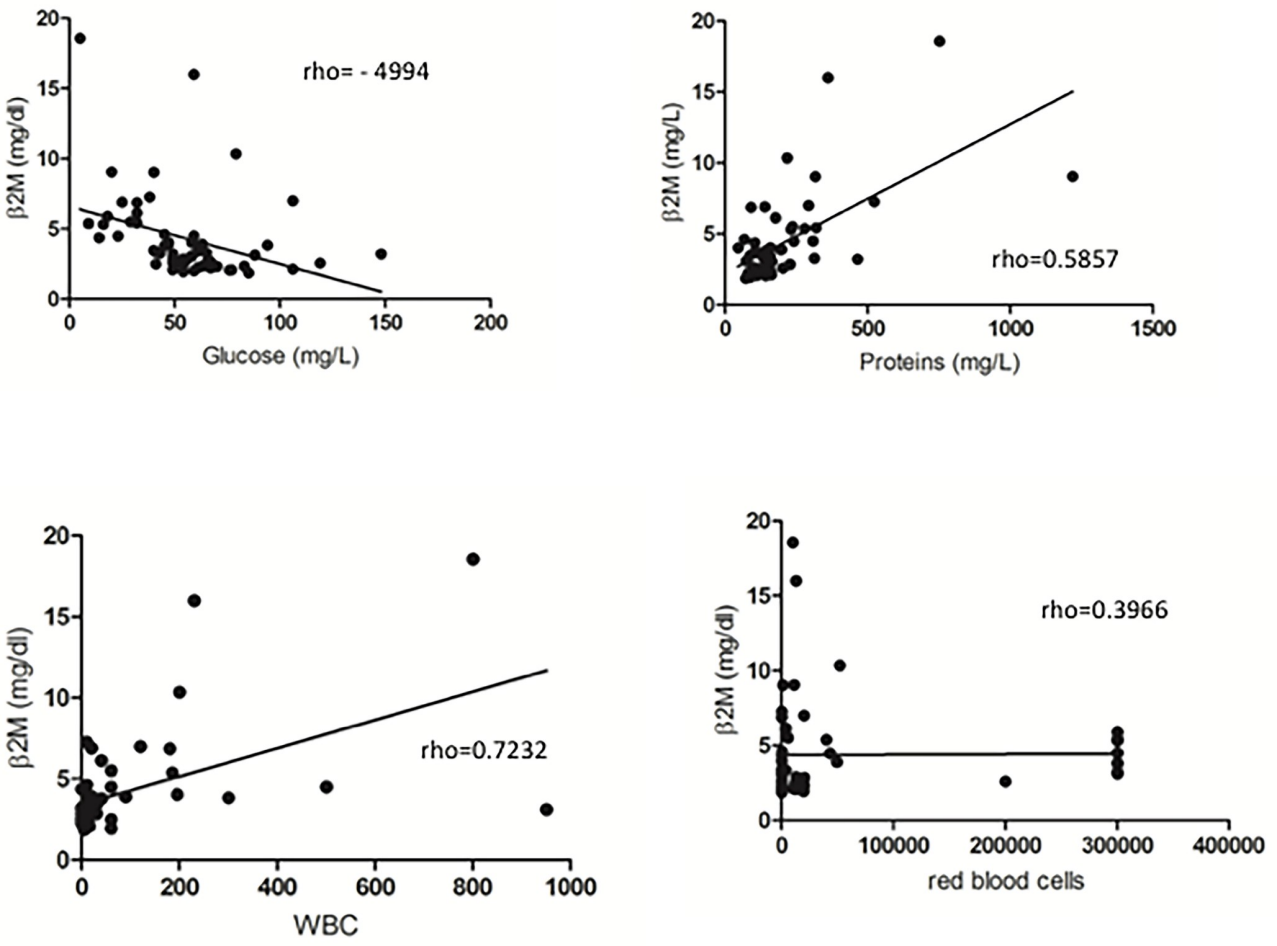
Correlation between B2M and classical CSF biomarkers (WBC, proteins, glucose and RBC).

In the CSF, B2M had a strong direct correlation with WBC (rho = 0.7237; p = 0.0001) and a moderate direct correlation with protein levels (rho = 0.5857; p = 0.0001). There was a moderate inverse correlation between B2M and glucose (rho = -0.4994; p = 0.0001). There was a weak direct correlation between B2M and RBC (rho = 0.3866; p = 0.003) (*Fig 1*).

### Results in each study group

#### Control group

36 patients were included in the control group. Patients in this group included those with a “suspected infection” (n = 7), “infection without culture confirmation” (n = 10) and “sepsis with positive blood cultures but without CNS involvement” (n = 19).

Median levels of B2M were 2.45 mg/L (2.1–3.14), 2.64 mg/L (1.87–3.47) and 3.05 mg/L (1.16–4.63) in the three sub-groups respectively, without statistically significant differences. There were no differences in protein, WBC or glucose levels amongst these sub-groups.

#### Meningitis group

There were 9 patients in the meningitis group, 5 of them with a positive culture or positive viral detection (*Klebsiella oxytoca*, *Escherichia coli*, *Serratia marcencens*, *CMV*). The mean level of B2M in this group was 9.03mg/L (5). The mean WBC level was 2263.33/mm^3^, and the mean glucose level was 34.2mg/dL (28.2).

#### PHVD dilatation group

14 patients were included in the PHVD group. Each of these patients had an elevated CSF RBC level as well as a considerable IVH, confirmed by ultrasound. Levels of WBC and proteins were increased in comparison with the other groups. Mean B2M levels (5.53mg/L) were also elevated, with the highest levels in those patients with a ventriculostomy catheter reservoir.

These results are shown in *Table 2.*

### CNS inflammation

In order to evaluate whether or not B2M is a useful tool to identify patients with CNS inflammation (including those with a CNS infection or PHVD), the main biochemical parameters in CSF were analyzed.

WBC, the most commonly used parameter to identify patients with CNS inflammation, had a relatively low sensitivity of 77.27%, despite a high specificity of 100% (AUC = 0.8955). Using a ROC curve, we determined the optimal cut-off point for the detection of CNS inflammation to be 25 WBC/mm3. Overall, the WBC count was not the most effective marker of CNS inflammation.

B2M as an indicator of inflammation had a sensitivity of 86.96% and a specificity of 94.44%, with an AUC = 0.9330. The optimal cut-off point obtained using a ROC curve was 4.04 mg/L.

Sensitivity, specificity, positive predictive value, negative predictive value and false negative percentage of these parameters utilizing these cut-off values are summarized in *Table 3.*

**Table 3:** CNS parameters characteristics as inflammation and infections test.

Parameter	AUC	Cut-off	Se (%)	Spe (%)	PPV (%)	NPV (%)	False negative (%)
Inflammation							
WBC	0.8955	25 WBC/mm3	77.27	100	100	87.50	22.73
B2M	0.9330	4.04 mg/L	86.96	94.44	90.9	91.89	13.04
Infection							
WBC	0.9505	30	100	78.4	50	96,7	11.1
B2M	0.9938	3.92	88.89	100	100	97.30	9.26

B2M: beta-2-microglobulin; WBC: white blood cells; AUC: Area under curve; Se: Sensitivity; Spe: Specificity; PPV: positive predictive value; NPV: negative predictive value

Multivariable logistic regression analysis was used to assess the best predictive model for CNS inflammation. The combination of B2M levels and glucose was the best equation to predict inflammation (AUC = 0.9690, sensitivity = 85.71%, specificity = 96.67%, PPV = 94.74%, NPV = 90.62%), however, this combination was not significantly better than B2M alone (p = 0.2188). Other equations including WBC or protein levels did not improve the predictive model.

**Fig 2:**
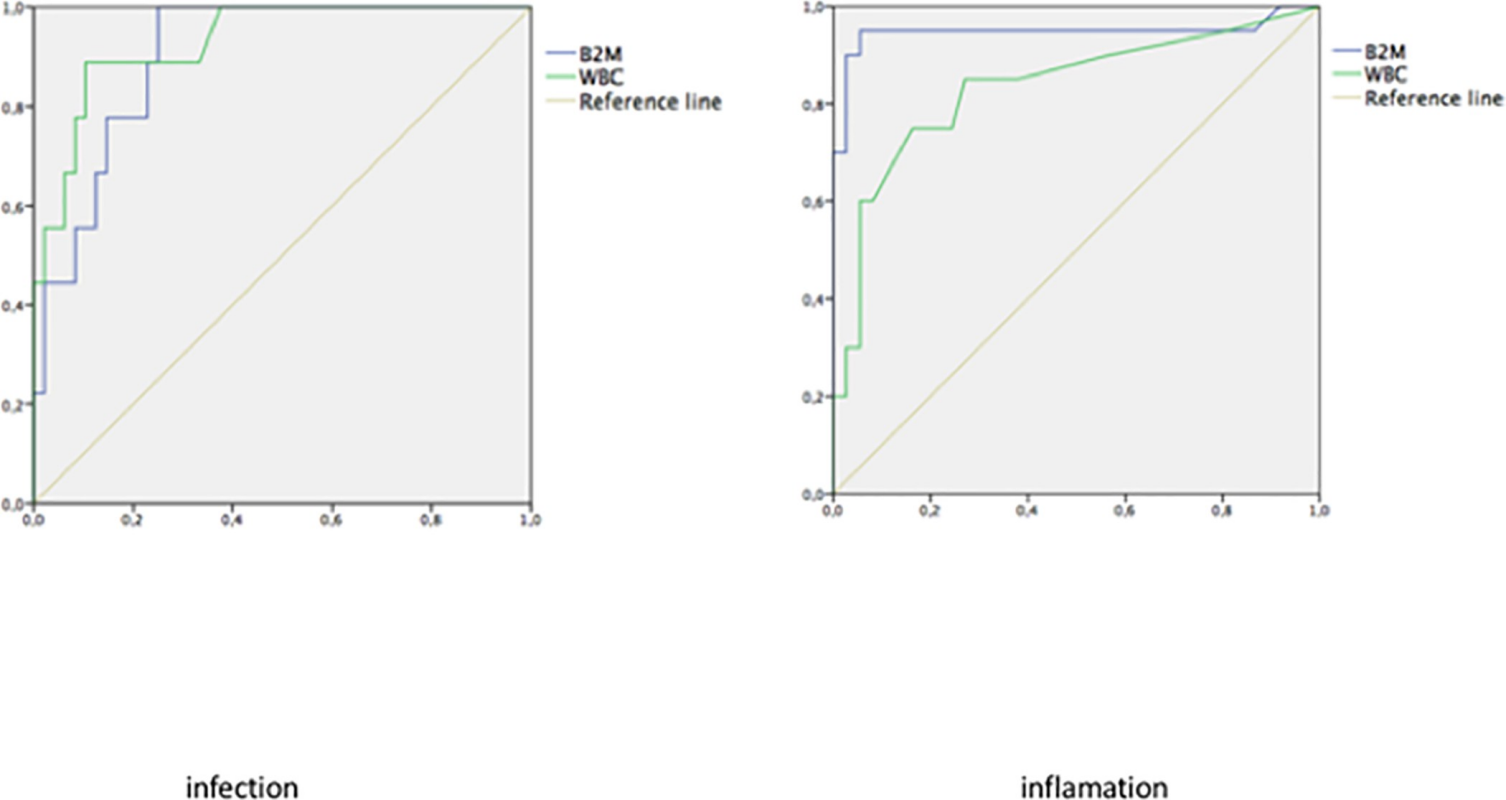
Prediction ROC curves: A/ infection: comparison of B2M curve (AUC = 0.99) with WBC curve (AUC = 0.95); B/ inflammation: comparison of B2M curve (AUC = 0.95) with WBC curve (AUC = 0.83).

ROC curves for CSF B2M levels and WBC as a means of screening for CNS inflammation are shown in *Fig 2*.

### CNS infection

A similar process was utilized to identify the best CNS infection biomarker. We excluded all patients with PHVD in the analysis to avoid confounding variables.

WBC as an indicator of CNS infection had a sensitivity of 100% and a specificity of 78.4%, with an AUC of 0.9505. The optimal cut-off value to diagnose a CNS infection was 30 WBC/mm3.

B2M as a biomarker for CNS infection had a sensitivity of 88.89% and a specificity of 100%, with an AUC of 0.9938. The optimal cut-off point to diagnose a CNS infection was 3.92 mg/L.

Sensitivity, specificity, positive predictive value, negative predictive value and false negative percentage of these parameters utilizing these cut-off values are summarized in *Table 3.*

Multivariable logistic regression analysis was also used to assess the best predictive model for CNS infection. The best equations to predict infection were the combination of WBC with B2M and B2M alone. The comparison of two ROC curves was not statistically different (*Fig 2).*

## Discussion

Early diagnosis of CNS infection is essential for prompt and optimal therapy. Acute infection requires rapid turn-around testing with a high predictive value, that is, the ability of a test to identify those patients who do and do not have a disease caused by a specific etiology.

CSF culture is still the gold standard method for diagnosing bacterial meningitis, but the culture can take several days to finalize. [4]. Moreover, some newborns are treated with empiric antibiotics before an LP can be performed, so the result of the culture may be falsely negative. For these reasons, the medical management of newborns with suspected meningitis is often based on clinical suspicion and biochemical analysis of the CSF [7].

These drawbacks are even more important in preterm infants. In this population, biochemical parameters of CSF are often difficult to interpret due to traumatic LPs, frequent antibiotic use and the higher incidence of IVH [6].

Normal values for CSF WBC, proteins and glucose are well established in full-term infants [5,6,16]. Kestenbaum et al showed in a study with 1064 infants younger than 56 days that newborns without bacterial meningitis had a median CSF WBC count of 3/mm3[5]. Martin-Ancel and colleges had similar results in a population of asymptomatic term newborns [16]. There is increasing evidence that there are no differences in WBC count and glucose levels in healthy preterm babies as compared to full-term infants. However, protein levels seem to be increased in the CSF of preterm infants [6].

Most clinical guidelines suggest using CSF WBC levels over 20–30 WBC/mm3 for the diagnosis of meningitis. This cut-off point has a sensitivity of 79% and specificity of 82% in term newborns [17,18]. In our population of very preterm and extremely preterm infants, the use of CSF WBC to diagnose meningitis had a great sensitivity (100%), but a low specificity (78.4%). It is important to consider that in clinical practice, an elevated CSF WBC is considered diagnostic for meningitis. As a result, there is a possibility of bias in the interpretation of these values. Furthermore, WBC counts in CSF can be falsely elevated due to traumatic LPs, which can make interpreting the data more difficult. In order to avoid false negative diagnoses, it seems reasonable and necessary to find other CSF biomarkers to diagnose meningitis, improving sensitivity and specificity.

Several molecules have been previously studied as potential bacterial meningitis biomarkers. The most sensitive ones described in the literature are cytokines such as IL-6, IL-10, TNF-alfa and INF-gamma, which are increased in the CSF of children with bacterial meningitis [8, 9,19]. However, routine measurements of these cytokines are not feasible in most hospitals.

B2M levels in CSF are increased in patients with neoplasia processes, CNS infections and other CNS inflammatory process [20, 21]. There is substantial evidence backing the potential utility of B2M as a CNS infection/inflammation biomarker [11]. *Garcia-Alix et al* reported reference values of CSF B2M in healthy term newborns in 1995 [13]. The same authors observed that these values were increased in babies with CNS infections [14]. An additional study described the utility of simultaneous evaluation of ferritin and B2M levels as an ancillary tool in the differential diagnosis of bacterial and viral meningitis [9]. B2M has also been reported to be increased in congenital CNS infections associated with neurological impairment [14, 22, 23].

Our study describes the concentration of B2M in CSF and its variation in different clinical situations in very preterm and extremely preterm infants. However, similar to other studies describing the normal cytochemical CSF values in newborns, patients in the control group were very preterm and extremely preterm infants with suspected and/or confirmed sepsis, given that it is not ethical to perform LPs on healthy infants [9,11,13].

Although we did not measure B2M levels in serum, we observed a significant increase in CSF B2M concentrations in patients with sepsis. This has been previously described in the literature [11,13] and is consistent with the theory that there is increased synthesis of B2M in the CSF in many inflammatory processes.

Another remarkable finding that supports the use of B2M as a CSF biomarker in this group of patients is the fact that it does not seem to be affected by traumatic LPs. While B2M levels were increased in patients with PHVD, this seems to be more related to inflammation from cell destruction rather than the presence of blood in CSF [5,7]. As reported in other studies, WBC is not a very sensitive parameter to diagnose meningitis in this population of patients [10]. Considering that there is not a negligible percentage of traumatic LPs in preterm infants [24,25], we conclude that the level of B2M in CSF may be an effective ancillary tool for diagnosing meningitis in the preterm population.

Another important and novel aspect of our work is the description of B2M values in patients with PHVD. In this group, B2M levels were higher than in the control group, but lower than in the CNS infection group. This is in line with many publications that suggest B2M could be a good inflammatory biomarker [9,11]. Although we did not have any patients with both a ventricular reservoir and a CNS infection, our hypothesis is that B2M would increase dramatically from the patient's baseline in this situation.

Finally, it is important to note that B2M is not a good tool to discriminate between infection and other forms of inflammation. This being said, the patient's clinical status and cranial ultrasound screening can assist in making an accurate diagnosis.

B2M in CSF proved to be an effective biomarker of CNS inflammation and infection in our population, although our study was retrospective in nature and included a small population with a high prevalence of CNS inflammation (31%) and infection (17%). The combination of B2M with other CSF biomarkers did not improve the ability of B2M to predict inflammation in this population. It would be beneficial to perform a prospective study in order to determine a better cut-off point for B2M that excluded all false negatives to improve meningitis diagnosis in very preterm and extremely preterm infants.

### Conclusion

B2M is significantly increased in the CSF of very preterm and extremely preterm newborns with cerebral infections and other CNS inflammatory processes, such as PHVD. It seems to not be altered by moderate amounts of blood in the CSF (up to 500 RBCs), which is especially important when considering a biomarker in newborn infants given the technical challenges of performing lumbar punctures in this population. B2M has the potential to aid in the early and accurate diagnosis of meningitis, which can result in more timely and appropriate antibiotic administration.

## Supporting information

S1 File S1_File. (XLS)
